# Research on Movement Analysis and Guidance in Dance Learning Based on Data Mining

**DOI:** 10.1155/2022/9327442

**Published:** 2022-09-19

**Authors:** Guangle Yin, Jing Liu

**Affiliations:** Music Academy, Henan Polytechnic, Zhengzhou 450046, China

## Abstract

In dance, we must understand the essential meaning of dance movements from the inside and express them on the basis of dance. Therefore, in the process of developing new dance teaching methods, it is necessary to improve the basic education of dance students, so that they can express the emotions conveyed by dance through body language and movements, and improve dance expression ability. In this context, we made the research and reached the following conclusions: (1) the number of frames of different dance types is also different, and the number of frames to be learned is also increasing. The dance with the highest number of frames is Latin2, which has 3635 frames, and the dance with the highest number of frames that need to be learned is also Latin2, which requires 2519 frames to learn. (2) The data mining method is still the highest among the three methods, and the accuracy of the complete teaching method is 82%, which is the lowest among the three methods, and the accuracy of the decentralized teaching method is 87%. No matter in the test set or the mixed test set, the curve values of deep mining are very stable. First of all, human movements emphasize that in dance, the essential meaning of dance movements needs to be understood from the inside and expressed through the foundation of dance. Therefore, when developing new dance teaching methods, it is necessary to strengthen the basic dance training of students so that students can express the emotions conveyed by dance through body language and movements and improve their dance expression ability. We conduct research in this ecological environment. Different types of dance learning process using different frames, different types of dance in the algorithm transport have different recognition methods, using better and different algorithms can achieve the best performance. Both groups in the Hip Hop dance had a shorter average learning time than both groups in the Latin dance.

## 1. Introduction

For representing, processing, and extracting knowledge for a variety of applications from the ever-increasing accumulation of data, which made the pervasiveness of computers possible, perhaps inevitable, and built a general framework for inspection and classification methods. Provide simple examples to demonstrate the nature of representative feature selection methods, compare them using datasets with a combination of intrinsic properties according to the goal of selecting features, propose guidelines for using different methods in various situations, and identify areas of research new challenge venue. A reference for researchers in machine learning, data mining, knowledge discovery, or databases [1]. As the ability to track and collect large amounts of data using current hardware technologies continues to improve, there has been interest in developing data mining algorithms that protect user privacy. A recently proposed technique addresses the privacy preservation problem by perturbing the data and reconstructing the distribution at the aggregation level to perform mining. This approach preserves privacy when accessing information implicit in the original attributes. The distribution reconstruction process naturally leads to some information loss, which is acceptable in many practical situations, and the algorithm is more efficient than currently available methods in terms of information loss levels. Specifically, it is demonstrated that the EM algorithm converges to a maximum-likelihood estimate of the original distribution based on perturbed data. When large amounts of data are available, the EM algorithm provides robust estimates of the raw data [2]. Interest in mining time series data has exploded over the past decade. In this work, the following claims are made. Much of the utility of this work is small because the amount of improvement provided by the contributions is entirely the variance that can be observed by testing on many real-world datasets, or by changing minor implementation details. To illustrate the point, the most exhaustive time series experimental reimplementation ever performed [3]. When used with statistical methods, graphical models have many advantages in data modeling because the model encodes the dependencies between all variables, can easily handle missing data, and can be used to understand problems and predict consequences. The article discusses methods for building networks from prior knowledge and summarizes Bayesian statistical methods for improving these models using data [4]. Using soft computing provides a survey of the available literature on data mining. Classifications have been provided based on the different soft computing tools used and the data mining functions they implement and the data mining functions implemented and preference criteria selected by the model. The utility of different soft computing methods is highlighted. Generally fuzzy sets are suitable for dealing with problems related to pattern comprehensibility, mixed media information and human interaction, and can provide approximate solutions faster. Genetic algorithms provide efficient search algorithms to select models from mixed media data, pointing out some challenges to data mining and applications of soft computing methods are presented. An extensive bibliography [5] is also included. Girls showed a higher personal interest in dance than boys, but the two groups were equally interested. Although girls were not as physically active as boys, their skills/knowledge outcome indicators were higher than boys. It appears that gender has little effect on the motivational effect of situational interest, and the quality of classroom learning in girls may be higher than in boys due to higher personal interest. The findings suggest that situational interest may motivate all students, but it is necessary to enhance personal interest in order for them to engage in high-quality learning [6]. We emphasize generating augmented reality environments for individual dancers based on dance annotation analysis. We have introduced a new interactive technique for dance animations required for educational purposes. This approach opens up new possibilities for interactive dance observation, fast and slow motion, different perspectives, and multiple functions to support high interactivity between users and 3D dancers [7]. All people have the urge to express themselves through dance, which represents a unique opportunity to artificially capture human creative expression. In particular, the spontaneity and relative ease of moving with music without any overall planning suggests a natural link between temporal patterns and motor control [8]. Four preschool dance classes, including 32 children aged 3–6, were randomly assigned to pretend imagination and traditional instruction. Ratings were based on the speed at which new motor skills were learned, future recall of the skills, concentration on the task, and enjoyment during the task. It was found that children in the pretend imagination group had significantly better visual fixation, engagement, and enjoyment of the task process, required less time for prompting and recall, and took less time to learn skills [9]. These multimedia applications need to store and retrieve different forms of media such as text, hypertext, graphics, still images, animation, audio, and video. Dance is one of the important cultural forms of a nation, and dance video is such a multimedia form. Archiving and retrieving the required semantics from these dance media collections are a crucial and demanding multimedia application [10]. In recent years, with the increase of work pressure, more and more people have begun to pay attention to national fitness, in order to improve personal physical and psychological quality. Square dancing is a sport suitable for all ages. It integrates fitness, a healthy heart, and a strong and handsome brain. It is the best way to communicate in fitness and is deeply loved by people. The current situation of residents' square dance was investigated, and the characteristics of square dance practitioners themselves and the characteristics of square dance were analyzed [11]. Track their movements using an optical motion capture system. Many pose and kinematic features are extracted from motion data. Findings indicated that the positive effects of proximity motivation were associated with higher movement speeds of the hands and head, as well as the extended posture of the hands. In addition, positive effects were also associated with higher-dimensional movements, suggesting an association with more complex and varied dance movements [12]. Chinese classical dance steps with unique aesthetics are closely related to traditional Chinese opera. Classical Chinese dance steps can be traced back to ancient women's foot binding, which had a great impact on the physiology and morphology of the dance steps [13]. Develop a method for automatic classification of the correctness of basic dance movements to support dance beginners in their self-practice outside the classroom. We first worked with dance professionals to design two basic dance moves, the waltz and the merengue. Acceleration and angular velocity data of the chest and pelvis were collected [14]. The article provides information on the health benefits of several dances. It mentions that dancing is a weight-bearing exercise that causes osteoblasts to grow. It added that dancing like boogie can enhance brain power, especially mental function and creativity. Furthermore, it mentions that dancing, including spinning, also burns calories, which is essential for a healthy heart [15].

## 2. Dance Learning

### 2.1. Status Quo of Dance Learning

#### 2.1.1. Classroom Learning Mode Is Single

Because the basic level of dance of the students varies, it is difficult to carry out high-level teaching, and only the intermediate level can be unified and standardized. Students with a good foundation feel that it is “tasteless to eat, and it is a pity to abandon it,” and repeating the content they have learned in the past feels a little dull and long; while students with a weak foundation are “dazzled” by the basic content and “hesitant” for the advanced content. In actual learning, teachers lead students to count the beats and jump over and over again, and “fill-in” mechanical imitations cause students to lose their enthusiasm for autonomous learning. The grasp of dance style is submerged in form, which restricts students' innovation and creativity.

#### 2.1.2. Curriculum Setting Is Biased toward Professional Technical Training

Although the construction of dance teaching materials tends to be perfect, the curriculum covers a wide range, and professional theoretical courses such as Chinese dance history and Chinese folk dance culture have not been properly used in teaching. Just by simply sorting out and forcing memory, students cannot think about the connotation and value of their cultural system and bring them into the process of dance performance.

#### 2.1.3. Lack of Distinctive Course Content Settings

The content and methods of dance course materials in advanced colleges have been tested by long-term teaching practice and tend to be stable, perfect, and self-contained. Different schools have different school-running characteristics. If the theory is blindly copied and the exploration of teaching practice is ignored, the content of the courses is similar, and there is a lack of regional targeting. In this way, the theory and practice of teaching are disconnected. For example, in the ethnic dance courses in the southwest region, if the advanced dance courses in other regions are set up step by step, there will be very few professional practice opportunities for students after graduation, which is not conducive to the study of ethnic culture and the inheritance of art in the southwest region. The teaching effect is maximized.

### 2.2. Problems in Dance Learning

#### 2.2.1. The Lack of Exemplarity in Dance Education

If there is a need, there will be a market, and schools will slowly emerge to teach dance. Whether it is teaching, teachers, or educational institutions, the conditions are relatively comprehensive, and the social recognition is relatively high, but more and more dance schools are social institutions that lack education and teaching. In response to this problem, the head teacher asked teachers to train students according to the grade-level learning materials and prepare for the exams that the students will take. As the class progresses, children are the main source of students in dance schools. Students in this age group are special because they are more playful, have shorter attention spans, and do not understand many technical terms. Many teachers do not pay attention to the details of students' physical and mental development. This creates a vicious circle. Finally, some dance schools only shout slogans and do not provide a good learning environment. The poor quality of teaching materials is not suitable for teachers. They always use a variety of teaching methods without caring about what each student's dance really means.

#### 2.2.2. The Teaching Method of Dance Education Is Not Systematic

**Figure 1:**
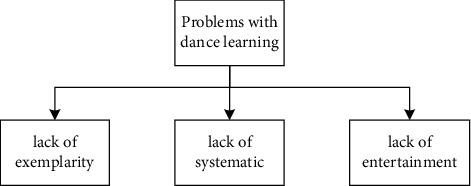
Problems in dance learning.

The teachers of some dance schools are not of high quality and cannot arrange courses according to the learning ability of students of different age groups. In order to make learning more effective, they blindly teach students difficult dance moves, ignoring the rules of easy and difficult to learn. Exceeding a student's physical capacity can affect not only his or her long-term physical development but also many potential risk factors. Others say learning to dance is difficult. Only strengthen the physical development of students. The intensity of the internship provides good grades but does not allow for dangerous movements that exceed the student's physical endurance. Doing too much to achieve course goals in a short period of time may be detrimental to the healthy development of students. Additionally, many teachers are unable to teach students based on information about the learning process. For example, some students have poor physical fitness and poor dance skills; so teachers have to learn to be careful not to trust each other, so that students eventually lose confidence in learning dance. It is shown in Figure 1.

### 2.3. Measures to Solve the Problems Existing in the Current Dance Learning

#### 2.3.1. Build an Excellent Team of Teachers

Everything related to education is inseparable from a good teacher. A good teacher must not only have good professional dance skills but also teach students moral education through language and action, that's how you can become a really good dance teacher.

#### 2.3.2. Pay Attention to Reasonable Arrangements for Teaching

Since most dance classes take place outside of school because of the need for concentration, teachers should give students enough time to learn about the teacher in class rather than just looking for a lot of knowledge. In addition, when explaining dance movements, teachers should try their best to explain in clear, clear, and easy-to-understand technical language to attract children's attention and stimulate their interest in learning.

#### 2.3.3. Strengthen the Construction of Hardware Facilities

It is necessary to strengthen the supervision of dance schools in all walks of life, and safety issues must be put in place in a timely manner, and dangerous accidents should be prevented as much as possible. Dance schools must determine that there is no income limit and no students will be admitted. This eliminates the need for quality teaching, liberating teachers, and increasing classroom efficiency.

#### 2.3.4. Strengthen the Construction of Dance Teaching Materials

**Figure 2:**
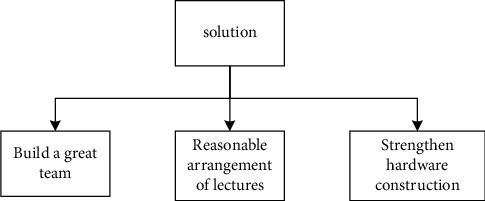
Measures to solve the problems existing in current dance learning.

Whether it is the Children's Palace or various extracurricular dance schools, the teaching materials need to be strengthened with the help of experts or experienced dance teachers to prepare the teaching materials for children's physical and mental development. Know how to do teaching tasks according to the actual situation of students in the classroom. Make a study plan and try to complete the study tasks correctly. Don't prepare for the exam. It is shown in Figure 2.

## 3. Data Mining Algorithms

### 3.1. Data Mining Algorithm Design

Create the subspace S required for data mining. Location is the value of the data function in the collection, and the data are in form *o* ∈ *D*. According to the data distribution characteristics of outliers, the nearest neighbors (*O, S*) of data elements can be expressed as also Subspace with uneven distribution. Regarding the nature of multidimensional data, it is clear that the focus of the subspace is the data object *O*, and the formula for calculating the probability distance is the following:

(1) $$\begin{array}{c} d_{s}=\frac{1}{I_{d}\left(o,s\right)}\text{.} \end{array}$$

In the formula, distance is represented by *d*. If the data object is always in the middle of the excavation set, the mean or distance of the data can be calculated by formula:

(2) $$\begin{array}{c} \sigma \left(o,s\right)=\sqrt{\underset{x\in s}{\sum }d{\left(o,s\right)}^{2}}\text{.} \end{array}$$

Since local discrete data have nonuniform distribution conditions, the properties of discrete data must be represented by an approximation between the density of discrete data and the standard distance.

(3) $$\begin{array}{c} \lambda =\frac{I_{d}}{\sigma \left(o,s\right)}\text{.} \end{array}$$

The segregated asset is obtained from equation (3) and the result provides the desired distribution of segregated data within the region. Combined with the above feature search method, the data entropy information algorithm is used to extract the information needed to retrieve big data objects from the Internet. Observe the distribution of the given data x, and use the probability function of the *p* value to obtain the information element *E*(*x*) of the data *x*:

(4) $$\begin{array}{c} E\left(x\right)=-\underset{x\in x}{\sum }p\left(x\right)\ln p\left(x\right)\text{.} \end{array}$$

Due to the differences in the detected data dimensions, it will have a certain negative impact on the results of IoT big data mining, so it is necessary that the detected data are processed according to the standard format, as shown in

(5) $$\begin{array}{c} \alpha =\frac{\alpha -\alpha_{1}}{Y_{\alpha }}\text{.} \end{array}$$

The result of data normalization should be calculated based on the average attribute of the received data and the data standard deviation attribute. The calculation process uses standard deviation to increase the relevance of the data properties and to ensure the accuracy of the data retrieval.

The overall results of data processing should be calculated according to the method, the nature of the data observed, and the standard deviation of the data characteristics. The calculation process uses the standard deviation to make data attributes more important to the accuracy of data mining. In addition, mean deviations from known data characteristics can also be used to obtain standard data processing results. The calculation formula is the following:

(6) $$\begin{array}{c} \alpha_{i}=\frac{\alpha -\alpha_{1}}{G_{a}}\text{.} \end{array}$$

The purpose of improving the anti-interference performance of the algorithm is achieved through the above formula, and the calculation formulas of the data attribute average *α*, the data attribute standard deviation *Y*_*a*_, and the data attribute average deviation *G*_*a*_ are the following:

(7) $$\begin{array}{c} \alpha_{1}=\underset{m}{\sum }\alpha \frac{1}{m}\text{,}\\ G_{a}=\underset{m}{\sum }\frac{\alpha -\alpha_{1}}{m}\text{,}\\ Y_{a}=\sqrt{\underset{m}{\sum }\frac{{\left(\alpha -\alpha_{1}\right)}^{2}}{m}}\text{.} \end{array}$$

In the formula, the number of iterations is *m*. After the data standardization is completed, artificial intelligence technology is applied to the processed data to obtain certain data mining results.

### 3.2. Realize Intelligent Data Mining

Neural network technology, an important branch of artificial intelligence technology, is used to carry out big data mining on the Internet of Things. The BP neural network with three-layer transmission structure is used as the main structure, and the normalized data are distributed into the neural network. Due to the particularity of the neural network structure, the average value of the information entropy of the data is *E*:

(8) $$\begin{array}{c} \omega =\frac{1-H_{i}}{\overset{E}{\underset{i=1}{\sum }}H_{i}}\text{,}\\ \theta_{1}=u\theta_{1}+\left(1-u\right)\theta_{2}\text{,}\\ \theta_{2}=u\theta_{2}+\left(1-u\right)\theta_{1}\text{.} \end{array}$$

The two data in the formula and *θ*_1_, *θ*_2_are linearly combined. The constant range of *U* is from 0 to 1, and the value range is narrowed according to the actual situation. If the constant value is fixed, it means that the hybridization operator is not constant during the calculation process. Since the constant value changes with the number of iterations, the average performance of the hybrid operator can be improved, and thereby realizing the integration of IoT big data.

(9) $$\begin{array}{c} v_{k}=v_{k}=\Delta \left(t,v_{k}-LB\right)\text{.} \end{array}$$

The data change value is generated from the left and right neighbors LB and UB of the variable *k* and the return value of the function. As the algebraic *t* increases, the value of the data change is likely to approach 0. Based on the above steps, a general operator search is performed on the dataset to create an IoT data view that meets the needs of data mining. In all the above processing steps, an IoT data mining algorithm based on artificial intelligence technology is developed.

### 3.3. Federated Average Algorithm

A unified learning algorithm distributes learning tasks to different devices to learn local model updates and, occasionally, interacts with a central server to coordinate learning objectives around the world. Unlike traditional machine learning methods, blended learning requires centralizing training models on a computer or data center so that each client device can use a local training data set to update the model. Federated learning is defined to solve the minimum loss of an objective function according to a formula.

The unified learning algorithm distributes learning tasks to different devices to learn local model updates and communicates with a central server from time to time to coordinate global learning goals. Unlike traditional machine learning approaches, blended learning requires training models to be centralized on a computer or data center so that each client device can use a local training dataset to update the model. Equation analysis is defined as the concentration of the dataset in a set of loss functions, which determine the solution to which Equation (10) is applied to the *k* samples of the dataset. The built-in analysis algorithm uses the generic, *n*=Σ_*k*_*n*_*k*_, *p*_*k*_=*n*_*k*_/*n* algorithm trained on all *k* multifunctional devices in the dataset.

(10) $$\begin{array}{c} \min f\left(\omega \right)=\frac{1}{n}\sum_{k=1}^{k}n_{k}F_{k}\left(\omega \right)=\sum_{k=1}^{k}p_{k}\left(\omega \right)\text{.} \end{array}$$

The local prediction loss function for each client device *k* along the model parameters is *F*_*k*_(*ω*). As shown in Equation (11), when different local optima are computed they are combined to achieve an overall minimization of the learning loss:

(11) $$\begin{array}{c} F_{k}\left(\omega \right)=\frac{1}{n_{k}}i\in n_{k}f\left(x_{k;}\omega \right)\text{.} \end{array}$$

This will improve common patterns in connected learning systems. To solve the objective equation, FedAvg first randomly selects *k* units from a subset of system units at each computational iteration, and then uses a combined FedSGD optimization method to globally implement each selected unit, computing the location of the global state and local data.

During the calculation process, local search and global search are continuously amplified according to P1 and P2 points, which correspond to both yin and yang. If point P2 is better than point P1 the two points are exchanged, and the sympathy algorithm agrees. Yin and yang have repetitions according to P1 and P1 points. P2 exploration optimization is expected to achieve a balance between local development and global exploration, which is a balance of yin and yang. In this particular implementation, the yin–yang balance optimization algorithm typically consists of two steps: updating the solution based on the collection set and updating the solution based on the hypersphere the main content of these two terms is given as follows:

(12) $$\begin{array}{c} g_{k}=\Delta F_{k}\left(\omega_{k}\right)\text{,}\\ \forall k,\omega_{t+1}^{k}⟵\omega_{t}-\eta g_{k}\text{.} \end{array}$$

In each of the update round, the server collects training data from all devices participating in that training round of the pattern and calculates the weighted average system update pattern according to equation (13), where *η* represents the training learning rate:

(13) $$\begin{array}{c} \omega_{t+1}⟵\omega_{t}\eta \sum_{k=1}^{k}\text{.} \end{array}$$

The number of elements in the FedAvg algorithm plays a crucial role in the speed of convergence and the accuracy of predictions. Some people suggest adding the stack size to some value to approximate disk. When installing a large compressed package, each device can speed up the calculation of the same amount of data, improve the convergence speed of the system, reduce the number of iterations, and keep the training of the system at a high enough level of accuracy. In the calculation process, the local search and global search based on points P1 and P2 are continuously strengthened, which corresponds to both yin and yang. If point P2 is better than point P1, then these two points are exchanged, and the sympathetic algorithm corresponding to yin and yang is repeated based on points P1 and P1. The optimization search of P2 is expected to achieve a balance between local development and global search, corresponding to the balance of yin and yang. In the specific implementation, the yin–yang balance optimization algorithm mainly includes two stages: solution update based on archive set and solution update based on hypersphere. The main contents of these two stages are given below. To update the search radius, the calculation method is as follows:

(14) $$\begin{array}{c} \delta_{1}=\delta_{1}-\left(\frac{\delta_{1}}{\alpha }\right)\text{,}\\ \delta_{2}=\delta_{2}+\left(\frac{\delta_{2}}{\alpha }\right)\text{.} \end{array}$$

## 4. Research on Action Analysis and Guidance of Data Mining in Dance Learning

### 4.1. Performance Test of Data Mining in Dance Movement Learning

**Figure 3:**
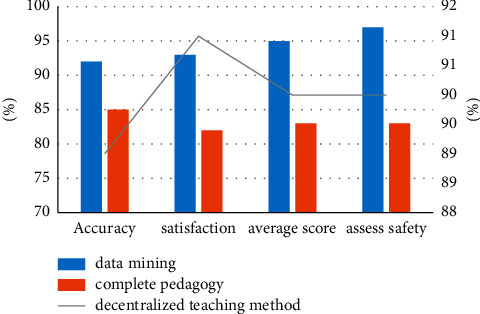
Data analysis on the test set.

To test the optimal performance of the data mining methods proposed in this dance review article, the models proposed in this article have been experimentally improved and research methods have been applied with subsequent tests and trials to make a complete and decentralized learning approach. Test kits are used to assess the generality of the final sample, and the hybrid test set meets the modern criteria of the model and is first used to assess the model's ability to write test results. Experimental data of different samples of the test group and the mixed test group are shown in Figure 3.

From the data in Figure 3, we can see that after performing a series of tests, the accuracy of the distributed learning method can be 89%, the accuracy can increase to 91%, and the accuracy can increase to 92% using data mining technology. The accuracy can increase to 93%, which is the highest value in the three test models. The complete teaching method has an accuracy rate of 85%, and the lowest of the three models. We can also see that the curve values of data mining technology are very stable, and the curve value of the decentralized teaching method remains around 0.90. The curve value of data mining is also always kept at 0.97, and the curve value of the complete teaching method is lower.

**Figure 4:**
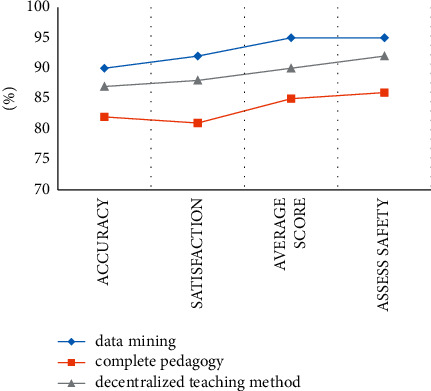
Data analysis on hybrid experience.

The data in Figure 4 and Table 1 shows that the performance of all three models decreases slightly after passing the mixed test series, but the data mining method proposed in the paper is still the highest of the three. The depth extraction curve is very stable whether it is a test series or a mixed test series.

### 4.2. Research on Movement Analysis and Guidance in Dance Learning

Based on the three aspects of human body structure, movement direction, and movement effect, the dance movement is reconstructed, the continuity and periodicity of dance movement data are determined, and a short and easy-to-understand language is developed to formally describe the MDL and application of dance movement. The structure of the MDL can be adjusted. As a description, language captures an individual's three-dimensional skeletal hierarchy, which is then described based on quantitative assessments of dance movements and biomechanical parameters (Tables 2 and 3). The action analyzes the spatial orientation of the human body, calculates the parameters of the movement characteristics from the key frame data, uses the description language to determine the movement trajectory of the human body, determines the correlation between the human body coordinates, and the spatial direction of the environment, and determines the description language to the human body movement trajectory.

There are many types of basic movements in dance learning, such as kicking the swallow, chest and waist, squatting, lowering the waist, stepping down, and rubbing the floor. The number of movements for dance learning needs to be systematically trained: 8 times/group of kicking swallows for a total of 5 groups; 10 times for chest and waist/group for a total of 3 groups; 15 times for squatting/group for a total of 3 groups; 5 times for lower back/group A total of 4 groups were completed; a total of 3 groups were completed under the span of 30 s/group; a total of 5 groups were completed by rubbing the floor 20 times/group.

**Figure 5:**
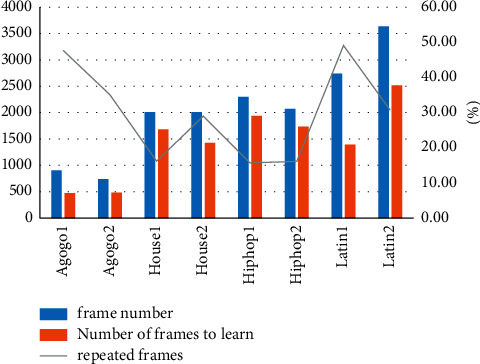
Number of repeating frames in dance.

From the data in Figure 5 and Table 4, we can see that the number of frames for different dance types is also different, and the number of frames to be learned is also increasing. The dance with the highest number of frames is Latin2, which has 3635 frames, and the dance with the highest number of frames that needs to be learned is also Latin2, which requires 2519 frames to learn. The dance with the highest number of repeated frames is Latin1 with 49.10% of repeated frames, and the dance with the lowest number of repeated frames is Hiphop1 with 15.70% of repeated frames. A total of students, including girls and boys, participated in our user survey. None of them had ever studied dance. We invite them to learn two dances, dance and dance. These students were divided into two groups, group and group study using the free-view method, and group study in the first stage of the course, as shown in Tables 5 and 6.

**Figure 6:**
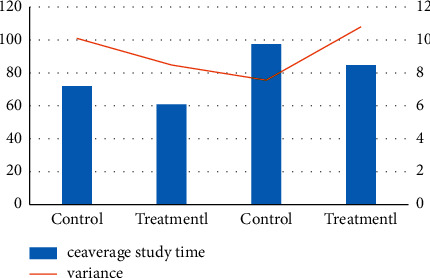
Statistics of the learning time of the control group and the treatmentl group.

From Figure 6 and Table 5, we see that the groups of students who learned to dance reported the average time spent (in minutes). The average learning time of the two groups of hip hop was 71.95 and 60.95, respectively, while the average learning time of the Latin group was 94.4 and 84.7, respectively. It can be seen that the average learning time of the two groups in hip hop dance is shorter than that of the two groups in Latin dance.

From the data in Table 7, it can be seen that the learning data of the two groups are very different (Hip hop : *T* = 3.74, *P*=0.0006; Latin: *T* = 4.3, *P* < 0.0001). Therefore, we believe that the first stage of dance teaching two this dance is more effective than the free-view method, so it works well.

**Figure 7:**
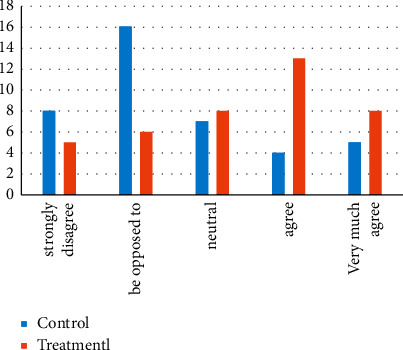
Comparison of control group and treatmentl group.

Given the data of the two groups of students on the questionnaire question “I think this system can help me learn dance,” from the experimental data in Figure 7, we can see that the proportion of those who hold opposing opinions is the largest in the control group. 16 people. In the Treatmentl group, the maximum proportion of people who agree with it reaches 13 people. It can be seen that there are more people in the control group who disagree with the Treatmentl group.

## 5. Conclusion

There is no direct connection between human movement and dance. In dance, the focus is on the movements of the people. The importance of dance moves must be understood from within and manifested on the basis of dance. Therefore, when developing new dance teaching methods, students need to improve their basic dance practice so that they can express the emotions conveyed by dance through body language and movements. In order to achieve the increase of dance performance. The goal of dance itself is to use the students' internal motor activities to enable them to achieve a double improvement in movement and expression.

## Figures and Tables

**Table 1:** The performance of each method on the mixed test set.

Method	Accuracy (%)	Satisfaction (%)	Average score (%)	Assess safety (%)
Data mining	90	92	95	95
Complete pedagogy	82	81	85	86
Decentralized teaching method	87	88	90	92

**Table 2:** MDL action description description.

Main content	Variable type	Variable name	Variable description
Basic information	RATE	m_rate	Action collection frequency
Structure	BODY	m_body	Body parts
Position	LEVEL	m_level	Horizontal orientation
Power effect	BEND_LEG	m_legbending	The degree of bending of the legs

**Table 3:** Movement analysis in dance learning.

Dance moves	Number of actions	Number of action groups
Kick the swallow	8 times/set	5 groups
Chest and waist	10 times/set	3 groups
Squat	15 times/set	3 groups
Waist	5 times/set	4 groups
Down span	30 s/set	3 groups
Rub the ground	20 times/set	5 groups

**Table 4:** Number of repeated frames in dance.

Dance moves	Frame number	Number of frames to learn	Repeated frames (%)
Agogo1	903	472	47.70
Agogo2	740	480	35.10
House1	2008	1682	16.20
House2	2010	1427	29
Hiphop1	2300	1939	15.70
Hiphop2	2069	1736	16.10
Latin1	2738	1393	49.10
Latin2	3635	2519	30.70

**Table 5:** Grouping of students.

Dance	Group	Learning method	Number of students
Latin	Control	Browse freely	20
	Treatmentl	First stage course	20

Hip hop	Control	Browse freely	20
	Treatmentl	First stage course	20

**Table 6:** Statistics of learning time of control group and treatmentl group.

Dance	Group	Number of students	Ceaverage study time	Variance
Latin	Control	20	71.95	10.1
	Treatmentl	20	60.95	8.48

Hip hop	Control	20	97.4	7.56
	Treatmentl	20	84.7	10.8

**Table 7:** *t*-Test results of learning time in control group and treatment1 group.

Group	Ceaverage study time	Variance
Control	71.95	10.1
Treatmentl	60.95	8.48
Control	97.4	7.56
Treatmentl	84.7	10.8

## Data Availability

The experimental data used to support the findings of this study are available from the corresponding author upon request.

## References

[1] Parpinelli R. S., Lopes H. S., Freitas A. A. (2002). Data mining with an ant colony optimization algorithm. *IEEE Transactions on Evolutionary Computation*.

[2] Pal S. K., Talwar V., Mitra P. (2002). Web mining in soft computing framework: relevance, state of the art and future directions. *IEEE Transactions on Neural Networks*.

[3] Frank E., Hall M., Trigg L., HolmesWitten I. H. (2004). Data mining in bioinformatics using Weka. *Bioinformatics*.

[4] Yang Q., Wu X. (2006). 10 challenging problems in data mining research [j]. *International Journal of Information Technology and Decision Making*.

[5] Hastie T., Tibshirani R., Friedman J. H. (2009). The elements of statistical learning: data mining, inference and prediction. By [J]. *The Mathematical Intelligencer*.

[6] Shen B., Chen A., Tolley H., Scrabis K. A. (2003). Gender and interest-based motivation in learning dance. *Journal of Teaching in Physical Education*.

[7] Ofli F., Erzin E., Yemez Y., Tekalp A. M. (2012). Learn2Dance: learning statistical music-to-dance mappings for choreography synthesis. *IEEE Transactions on Multimedia*.

[8] Sacha T. J., Russ S. W. (2006). Effects of pretend imagery on learning dance in preschool children. *Early Childhood Education Journal*.

[9] Batcheller J. (2012). Learning how to dance: courageous followership. *Nurse Leader*.

[10] Wang Y., Liu Q. (2020). Effects of game-based teaching on primary students’ dance learning. *International Journal of Game-Based Learning*.

[11] Saarikallio S., Luck G., Burger B., ThompsonToiviainen P (2013). Dance moves reflect current affective state illustrative of approach-avoidance motivation. *Psychology of Aesthetics, Creativity, and the Arts*.

[12] Wulff H., Martin R. (1999). Critical moves: dance studies in theory and politics. *Contemporary Sociology*.

[13] Jong K. D., Amorim M., Fonseca P. J. (2019). Singing and dancing fish: females pay more attention to males’ dance moves when it is noisy [J]. *Frontiers for Young Minds*.

[14] Lee H. (2012). A study on analysis system on dance moves by applying munmyoilmu demonstrative illustrations [J]. *The Journal of Korean Dance*.

[15] Fontanesi C., DeSouza J. F. X. (2020). Beauty that moves: dance for Parkinson’s effects on affect, self-efficacy, gait symmetry, and dual task performance. *Frontiers in Psychology*.

